# A thin line between consent and abuse - Reflections on sexual experiences among Swedish young adults with intellectual disabilities

**DOI:** 10.1177/17446295241276245

**Published:** 2024-10-08

**Authors:** Anna Hart, Charlotta Löfgren, Charlotta Carlström, Eva Elmerstig

**Affiliations:** Centre for Sexology and Sexuality Studies, Sweden; Department of Social Work, Faculty of Health and Society, 5264 Malmö University, Sweden

**Keywords:** Intellectual disabilities, sexual consent, sexual experiences, sexual script, thematic analysis

## Abstract

Research shows that young adults with intellectual disabilities struggle to understand the social codes embedded in sexual situations. This may lead to an incomprehension of sexual consent, or when consenting to sex may lead to abuse. This qualitative study aimed to gain an in-depth understanding of sexual experiences and perceptions about sexual consent among Swedish young adults with intellectual disabilities. The data comprise 22 semi-structured interviews with young adults aged 18-35 with intellectual disabilities, thematically analyzed using sexual script theory as a theoretical framework. Four themes emerged from the analysis: Setting the sexual scene, Sexual self-awareness, Sexual communication and the necessity of clarification and Striving for a balance. The results show that the participants had a variety of sexual experiences and challenges associated with sexual consent, but also an agency that favors sexual situations. Further research is necessary to understand the complexities of sexual consent in this population.

## Introduction

This article focuses on challenges related to sexual consent among young adults with intellectual disabilities. Sexual consent in general is a dynamic and interactive process that can be difficult to navigate. Attaining mutual consent within a sexual context requires a multifaceted interaction that involves many social codes (Russell-Miller, 2020). This has been illustrated in a Swedish study of adolescents from the general population (Holmström et al., 2020). The adolescents discussed various aspects of consent, including the ways certain stereotypical scripts, such as going home with someone after a night out, could be interpreted as sexual consent. They also described ambiguities, such as whether giving consent once means per se that this consent is also present in the next sexual situation (Holmström et al., 2020). An international literature review of the general population indicates that sexual consent is normally communicated non-verbally (Johnson and Hoover, 2015). Still, the communication of verbal consent usually takes place in an indirect process, e.g., as an act followed by verbal clarification (Russell-Miller, 2020).

Research shows that the social codes embedded in sexual situations may be difficult for young adults with intellectual disabilities to interpret (Foley, 2018; Jahoda and Pownall, 2014; Stoffelen et al., 2018). This could lead to not knowing or understanding sexual consent or when consenting to sex can lead to abuse (Eastgate et al., 2011; Willot et al., 2020). For example, the findings of Eastgate et al., (2011) among women with mild intellectual disabilities revealed challenges in navigating intimate relationships. These challenges included having limited confidence and difficulty resisting unwanted sexual advances as a result of inadequate sex education and difficulties interpreting their sexual boundaries. Findings reported by Schaafsma (2013), who studied young adults with mild intellectual disabilities, revealed that they had limited social networks, reduced sexual experience, and limited knowledge about sexuality and relationships in comparison with the general population. Similar results have also been reported in more recent research (Baines et al., 2018; Löfgren-Mårtenson, 2020b; Retznik et al., 2021). This may have an impact on perceptions of sexual consent in sexual situations, but according to Kousmanen and Starke (2015) and Willot et al. (2020), it also relates to the cognitive difficulties that individuals with intellectual disabilities have in interpreting sexual boundaries and understanding whether they are being exposed to sexual violations. Recent research by Hollomotz et al. (2023) highlights the lack of adequate professional support, e.g., tailored communication for individuals with disabilities who have experienced sexual violence. In addition, as has been noted by both Schaafsma (2013) and Löfgren- Mårtenson (2020a), many do not know where to turn to obtain information about sexual health and rights. This may be due to several factors, including insufficient or ineffective communication and a tendency towards an overprotective approach from society towards the target group's sexuality, which is in turn due to for example, concerns about and a desire to prevent, sexual assault and unwanted pregnancies (Löfgren-Mårtenson, 2020a; McCarthy et al., 2022; Nayak, 2016). All this adds up, leading to a blurred understanding of sexual boundaries or sexual grey areas (Russell-Miller, 2020). These may influence the ability to understand and practice sexual consent, justifying research in this area.

The process of clarifying intentions and alternatives in a sexual situation is very complex. Research shows that many young individuals with intellectual disabilities have an awareness of what is considered appropriate sexual behavior, but not of how to acquire or maintain sexual relationships (Bernert and Ogletree, 2013; Dinwoodie et al., 2020; Frawley and Wilson, 2016; Löfgren-Mårtenson, 2020b). McCarthy's (2014) study involving participants with mild to moderate intellectual disabilities revealed that individuals with mild intellectual disabilities often navigate a complex position between the intellectual disability population and the general population. This position may influence their decisions regarding sexual relationships and risk-taking. Swedish research has shown that young adults with intellectual disabilities want better education on social codes in sexual situations to navigate sexual consent (Löfgren-Mårtenson, 2020b), which is consistent with findings from international studies (Onstot, 2019, Schaafsma et al., 2017; Stoffelen et al., 2018).

Despite professionals’ (service providers, teachers, researchers) and relatives’ insights regarding sexuality issues among young adults with intellectual disabilities (Baines et al., 2018; Hollomotz, 2012), their understanding of the way young adults’ practice sexual consent remains unexplored. Moreover, while the degree of intellectual disability may not always be the central focus when it comes to experiences of sexuality, the emphasis in this study lies on individuals' lived experiences and their ability to articulate and reflect on these experiences. This highlights the significance of a certain level of cognitive ability (Semple and Smyth, 2019).

### Swedish context

Consensual sex has been and continues to be widely debated in the Swedish research context (Holmström et al., 2020). This does not apply to individuals with intellectual disabilities however, and only a few studies have focused on this area (Kuosmanen, and Starke, 2015; Starke et al., 2024). According to Swedish law, consensual sex requires clear verbal or physical consent (SFS 2018:618). In 2018, the Swedish rape law was revised. The offense is now based on the absence of consent, whereas previously it required the use of force or threats, or the exploitation of a particularly vulnerable situation (Holmberg and Lewenhagen, 2020). This means a person must ensure the other's voluntary participation. Given the challenges individuals with intellectual disabilities already face in understanding their own and others' sexual boundaries (Löfgren-Mårtenson, 2020b; Willot et al., 2020), adapting to the amendment in the Swedish rape law may present some practical difficulties for this group.

Persons with intellectual disabilities are entitled to support to facilitate their integration into mainstream society under the Swedish Act on Support and Services for Persons with Certain Functional Impairments (SFS, 1993:387). This support aims to bring their living conditions into line with those of the general population. For the service user, the act provides personalized professional support from others to maintain their daily routines. But this constant need for support results in assumptions being made about them by others, with the result that their privacy and sexual autonomy are compromised, and sexual activity and privacy are often impossible (Löfgren-Mårtenson, 2020b; Nelson et al., 2020). Löfgren-Mårtenson (2020a) notes that Sexual and Reproductive Health and Rights (SRHR) are important concepts but are not used in practice because the Swedish SRHR strategy has not been adapted to meet the needs of individuals with intellectual disabilities to exercise choice, responsibility, and sexuality e.g., by including sexual orientation into a comprehensive and relevant sexual education or by recognizing this group as sexual agents (Gill, 2015; Löfgren-Mårtenson, 2020a).

In Sweden, sexual education has been a mandatory component of the school curriculum since 1955, irrespective of the type of education provided (Löfgren-Mårtenson, 2012), and the curriculum has recently been revised to include sexual consent (Planting-Bergloo, 2023). However, Lukkerz (2023) argues that individuals with intellectual disabilities do not have the same access to this education as the general population and teachers delivering sexual education frequently lack the necessary expertise in the subject matter they are expected to teach.

In combination, these factors could influence the understanding of sexual consent among young adults with intellectual disabilities. Despite emerging sexuality research that has included the perspectives of young adults with intellectual disabilities (e.g., Löfgren-Mårtenson, 2020b; Nelson et al., 2020; Retznik et al., 2021), a notable knowledge gap persists regarding the target group's own input into the concept of sexual consent, which has remained unaddressed.

### Sexual scripts

The sexual script theory of sociologists John Gagnon and William Simon (2017) provides a useful theoretical approach to understanding how people comprehend and describe sexual and non-sexual situations. Sexual script theory is based on the view that sexual activity is a social and learned behavior and that the multidimensional interactions involved cannot be explained solely as a biological phenomenon but must be viewed in terms of social and cultural scripts (Gagnon and Simon, 1986, 2017). These scripts are divided into three types, cultural, interpersonal, and intrapsychic scripts, that are intertwined with one another on different levels. The cultural level includes the general norms and values that are present in a society, while the interpersonal level scripts organize relationships between individuals, with sexual behavior being created in a particular situation and dynamic depending on the setting. Finally, intrapsychic-level scripts, which stipulate how an individual should act and react in a sexual situation, are based on the person’s inner fantasies, preferences, and desires (Gagnon and Simon, 1986, 2017). Löfgren-Mårtenson (2013) further developed our understanding of traditional scripts in her research on people with intellectual disabilities. Her research found that sexual scripts geared towards people with intellectual disabilities are more restrictive compared to the general population. For example, the protective behavior of care providers, based on their feelings of responsibility, may limit the ability of individuals with intellectual disabilities to explore and express their sexuality. Consequently, many young adults with intellectual disabilities may be prevented from engaging in romantic or sexual relationships, thus impeding their capacity to develop relationships and experience intimacy autonomously (Löfgren-Mårtenson, 2013).

Since many people with intellectual disabilities live with restricted scripts, these scripts can be a useful contribution to the analysis. Using sexual script theory, the different levels are used as a framework for analyzing participants' sexual experiences and understanding of consent.

### Objective and research question

This study aims to gain an in-depth understanding of sexual experiences and perceptions of sexual consent among young adults (aged 18-35) with intellectual disabilities. The specific research question is: In what ways do young individuals with intellectual disabilities describe and reflect on their experiences of sexual situations and sexual consent?

## Methods

### Recruitment, participants and data collection

To reach in-depth knowledge this study has utilized a qualitative research approach based on semi-structured interviews (Creswell and Poth, 2018). Drawing on insights from previous research highlighting the capability of young individuals with intellectual disabilities to participate in discussions on sexuality (Frawley and Wilson, 2016), this study avoided categorizing participants by the severity of their intellectual disabilities. Instead, the focus was directed at contextual factors to select participants based on their environment. The inclusion criteria targeted individuals with the linguistic and communicative skills required for interview engagement, which may be considered a strategic selection (Creswell and Poth, 2018). Participants were recruited from diverse settings, such as day activity centers and specialized upper secondary schools, ensuring the presence of individuals with adequate linguistic and communicative abilities for verbal interviews.

In total, 22 interviews (5 men and 17 women) were conducted in Sweden. Sexuality may constitute a sensitive research topic, and interviewing individuals with intellectual disabilities can be challenging, which made it important to address several factors (Hollomotz, 2018). The first author, who conducted all the interviews, has practical experience working with sensitive issues, such as sexual issues, with the target group. From an ethical perspective, individuals with whom the first author had been in contact within the clinical practice have been excluded, as being in a state of dependence (Godwill, 2015).

### Recruitment

The study included participants aged 18-35 years, with intellectual disabilities, who were able to express themselves verbally in Swedish or English. In addition, they should have had experiences of sexual situations. Traditionally, the term young adults apply to the period between the late teens and the 30s, but it is difficult to specify definitive age thresholds (Hudson, 2006). Since the development of individuals with intellectual disabilities can vary individually, the decision was made to include participants up to age 35. The study was granted ethical approval by the Swedish Ethical Review Authority (Record number 2021–04974), and participants were recruited from across Sweden during the year 2022. The recruitment process involved initially contacting eleven organizations, including day activity centers and specialized secondary schools, and three national associations and networks in the disability field. These organizations facilitated discussions on sexuality and consent as part of their activities. Two participants were recruited through a national association, while the remaining participants were recruited via organizations. All those recruited agreed to participate.

The gatekeepers, specific individuals within the organizational structure (Ahern, 2014), held various positions. Some were closely involved with participants, while others held managerial roles. These individuals supported the first author and served as gate openers, facilitating the recruitment of participants with effective communication skills in line with the chosen research method (Creswell and Poth, 2018).

The ambition was to provide information to potential participants and enable them to meet with the researcher on a separate occasion before the interview, to have an opportunity to ask questions. But this has only happened as a secondary effect when carrying out planned interviews. Subsequently, the researcher provided additional information at the interview. This created a snowball effect where some more participants were recruited. All the participants who agreed to participate went on to complete the interviews. This indicates a general interest from the participants to share their experiences.

Table 1 provides general information on the participants. Pseudonyms and approximate ages have been used to protect the identity of the participants.

**Table 1.** General information on the participants.

Participants fictional names	Accommodation	Locality	Marital status	Age	Children	Positive sexual experiences	Negative sexual experiences	Not sure if negative/ positive
1 Judith	own flat	urban area	single	30–35		x	x	
2 Felicia	service accommodation flat	urban area	sexual buddy	24–29		x	x	
3 Eric	own flat	urban area	single	24–29			x	
4 Daisy	own flat	urban area	married	30–35	x	x	x	
5 Tove	living with parents	rural area	single	18–23			x	
6 Elsa	own flat	suburb	single	18–23		x	x	
7 Lina	accommodated living	suburb	engaged	24–29		x	x	
8 Nellie	accommodated living	suburb	in relationship	30–35		x		
9 Britt	accommodated living	suburb	single	24–29		x		
10 Rosa	own flat	suburb	single	30–35		x	x	
11 Tom	living with parents	urban area	single	30–35		x	x	
12 Lisa	living with parents	urban area	single	30–35		x	x	
13 Siv	living with parents	urban area	in relationship	18–23		x		
14 Gunnel	own flat	urban area	in relationship	30–35		x	x	
15 Nick	living with parents	urban area	in relationship	18–23		x		
16 John	living with parents	urban area	single	18–23			x	
17 Per	living with parents	urban area	single	18–23		x		
18 Mona	living with parents	urban area	single	30–35			x	
19 Suzi	accommodated living	urban area	single	30–35			x	
20 Ruth	accommodated living	urban area	single	24–29		x		x
21 Bibbi	accommodated living	urban area	in relationship	30–35		x	x	
22 Kicky	living with parents	urban area	single	18–23			x	

Table 1 shows the heterogeneity among the participants. The cognition of the participants has not been categorized, although individual variation was noted, which was addressed in the interview situation. During the interviews, it was further noted that some participants were involved in disability rights or other organizations.

### The interview situation

Considering the participants’ varying communication skills and the topic of sexuality, providing extra clarification concerning the interview process and the sensitive subject matter has been central in both the recruitment process and during the interviews, in line with previous research (Hollomotz, 2018). Interviewing individuals with intellectual disabilities may present the interviewer with a range of challenges. For example, participants might give short replies, or be loyal to those supporting them (Hollomotz, 2018). Previous research among people with intellectual disabilities indicates the importance of the researcher ensuring that the participants have fully understood the implications of participation in a research project and of information being adapted to meet the individual’s needs (Caldwell, 2014; Hollomotz, 2018). Before each interview, participants were informed about the project and written, or verbal informed consent was obtained (verbal consent was documented by audio recording). Control questions were used to confirm participant understanding and voluntary participation (Hollomotz, 2018).

Following previous studies in this field (Frawley and Wilson, 2016; Löfgren-Mårtenson, 2020b), a semi-structured interview guide was employed to cover the project topic, focusing on sexual situations and experiences. This approach was chosen to maintain a coherent focus on the research objectives (Creswell and Poth, 2018) while allowing for flexibility in the interview situation to accommodate the specific needs and communication styles of the participants (Frawley and Wilson, 2016; Hollomotz, 2018). The flexibility of the interview structure not only reduced the likelihood of researcher bias but also created an environment conducive to participants articulating their reflections and experiences freely (Hollomotz, 2018).

Open-ended questions and interviewees' experiences with sexual health within the target group were used to accommodate participants' diverse understandings and encourage context-specific responses (Caldwell, 2014; Emerson, 2012). The interviews began with general questions such as *''Tell me about yourself''* and *''Are you currently in a relationship?*'' The participants’ responses then guided more specific questions from the interview guide regarding sexual experiences, for example, *“Would you like to tell me more about when you and Kalle have sex?”* or “*How do you know you want to have sex with Kalle*?”Building on previous research with similar groups (Hollomotz, 2018; Löfgren-Mårtenson, 2020b), feedback questions were included to assist the researcher in ensuring that the participants’ understanding was adequate. These questions were constructed to address the participants' needs, and they served as checkpoints to verify understanding and optimize communication. The participants’ responses were actively summarized during or immediately after the questions and at the end of the interview to promote mutual understanding and allow the participants to modify or elaborate on their answers.

### Data analysis

The audio-recorded interviews were transcribed verbatim and inserted into the qualitative data analysis software tool NVivo (Dhakal, 2022). Coding and analysis were based on Braun and Clarke’s (2006, 2022) guidelines for thematic analysis, using both an inductive and a deductive style, to address the research question (Bryman, 2016; Creswell and Poth, 2018). An advantage of this analysis is its flexibility, which is not tied to a specific theoretical framework (Emerson, 2012), making it appropriate for the interview method of choice (Bryman, 2016; Hollomotz, 2018). The coding process considered both latent and semantic codes, including how information was conveyed and the underlying nonverbal cues (Braun and Clarke, 2022; Trainor and Bundon, 2020). Initial themes were developed through a rigorous process of reviewing and refining codes (Braun and Clarke, 2022).

In the end, four themes emerged: *Setting the sexual scene* highlights the various challenges encountered by the participants when initiating sexual activity, with a particular focus on societal expectations and norms relating to sexuality and their impact on the issue of sexual consent. *Sexual self-awareness* emphasizes the importance of understanding and managing one's sexual codes and emotions, as well as the ability to give and receive consent in a sexual context. *Sexual communication and the necessity of clarification* involves the crucial aspect of understanding and respecting both one's own and one's partner's sexual boundaries and the significance of clear and open communication in sexual relationships. *Striving for a balance* encompasses the participants' efforts to navigate and find stability in their diverse sexual experiences and relationships, balancing autonomy, safety, and personal fulfillment. These themes are further analyzed through the lens of Gagnon and Simon’s Sexual Scripting Theory (2017), which encompasses cultural, interpersonal, and intrapsychic levels. Because being young and having an intellectual disability adds an extra dimension to consensual sexual activity, Löfgren-Mårtenson's (2013) restrictive scripts are valuable in the analysis. Findings have been discussed among the authors throughout the process to facilitate credibility in the analysis (Nowell et al., 2017).

Table 2 illustrates the analytical process.

**Table 2.** Analytical process.

Themes	Setting the sexual scene	Sexual self awareness	Sexual communication and the necessity of clarification	Striving for a Balance
Subordinate Themes	Having sex like others - obstacles or support	Interpretation of sensations	Sex and sensibility - communication of sexual consent	Pleasure, or broken consent
	Reasons for wanting sex	Intimate partner pleasure	Perception of boundaries	Ambivalence and confusion
	Desire and vulnerability affecting consent			The line breaks - sexual violence

## Results

This section is structured around the four themes which emerged in the analysis. All the results are based on participants’ own reflections about sexual experiences with another person.

### Setting the sexual scene

This theme addresses the challenges participants encountered when initiating and engaging in sexual activities, where external factors greatly influence experiences and sexual consent. Participants' sexuality was often public in the sense that they lived at home or in accommodated living, which affected their ability to engage in sexual encounters. The creation of private sexual spaces demanded considerable effort from many of the participants, requiring extensive preparation and planning. Numerous obstacles were encountered, and participants employed various strategies to navigate public visibility and supervision. In this process, the prioritization of safety and sexual consent often became secondary or were forgotten. The narratives shared by the participants may be viewed as examples of sexual scripts (Gagnon and Simon, 2017) and restrictive scripts (Löfgren-Mårtenson, 2013).

Engaging and actively participating in sexual activities may influence a person’s capacity for giving consent and can be related to the specific living situation and the presence of care providers. In the interviews, it became apparent that external factors affected the possibility for the participants to have sex with their partners. For instance, if both had intellectual disabilities, lived in separate accommodated housing, and wanted to meet, then this often required meticulous planning. Nor was this planning restricted to the two individuals who wanted to meet, it also meant planning arrangements between the individuals’ separate care and accommodation providers, which could sometimes lead to a conflict of interests, since staff were unable to provide support to the visiting partner. In sexual scenarios, people living with parents or in supported housing face particular challenges because their sexuality is more publicly visible, as they are always in the presence of caregivers or parents. These can be seen as forms of restrictive scripts (Löfgren-Mårtenson, 2013) and could mean that caregivers or parents have to prevent or approve sexual activities. Having an LGBTQ identity can mean that you are outside of a certain set of norms, and having an intellectual disability can make the restrictive scripts even more apparent. Elsa, whose parents had conservative views on sexuality was aware of these scripts and kept her sexual identity secret from her parents while she was in a same-sex relationship and living at home, and thus did not have sex at home. Elsa reflected on the situation:

My dad is very homophobic, so it was really difficult. Because I already knew when I was twelve, that I liked girls more. So, you could not speak with each other. I mean, I had a girlfriend, and I never took her home. We were always at her place because I felt my parents would be the same then.

Elsa later had a violent heterosexual relationship where the value of parental monitoring became evident. At the time, Elsa's parents were at home, which Elsa found reassuring. They heard and intervened in the sexual assault, thereby demonstrating care providers' pivotal role in safeguarding individuals with intellectual disabilities while respecting their autonomy. To address the sexual aspect of the situation, where the presence of parents or care providers is a constant factor in the lives of the participants, some of the participants employed certain strategies for dealing with obstacles, such as putting up a “Do not disturb” sign on the door. Lina exemplified:

But if we have sex or are private, they always knock. I mean, I have a sign “do not disturb”, “busy” or something like that, so they don’t come in when I am intimate with my boyfriend.

It became apparent that the sexual scene in which various sexual activities took place affected the possibilities for young adults with intellectual disabilities to consent sexually. This became particularly apparent because there was so much more to consider even before being in a sexual situation. At the same time, the results show how individuals with intellectual disabilities can be sexual agents and set their sexual scene by e.g., leaving a message on the door.

Creating and maintaining a private and sexual sphere required a great deal of focus, so sexual consent and trust seemed to become secondary. Bearing in mind the way all the factors described above are involved in creating the sexual scene, the next theme looks more closely at how the participants described their sexual self-awareness in sexual situations.

### Sexual self-awareness

To engage in sexual situations, young adults with intellectual disabilities navigate complex parameters. These include interpreting their social codes and cues, as well as those of their partners. Making decisions about sexual consent may be difficult and take longer because of these factors. Paying attention to how participants presented themselves in sexual situations and how this affected sexual consent can provide insight into their sexual self-awareness. The ways in which they presented themselves in sexual situations showed that this self-awareness could differ depending on their sexual knowledge and life experiences.

For some participants, cognitive difficulties impeded the sexual consent process, particularly when interpreting sexual arousal cues. Challenges included difficulties in understanding and interpreting their own sexual body signals promptly, which affected their ability to establish consent in sexual situations and to renegotiate consent when necessary. For instance, some described difficulties in withdrawing consent during sexual activity after initially consenting. Conversely, others described how arousal was linked to verbal cues, resulting in immediate consensual sexual activity. For young adults with intellectual disabilities, this process is particularly complex. The intrapsychic script is closely linked to what feels good or bad in a situation, activating memories and emotions from past experiences. But it also relates to cultural and interpersonal scripts (Gagnon and Simon, 1986, 2017) and how they interact and sometimes collide, which may lead to misunderstandings and negative experiences although sometimes they are made understandable by common cultural frames of reference. Lina, who was in a new relationship and had previous experiences of sexual abuse, reflected on sexual self-awareness during the interview:

Interviewer: Could you tell me more about when you have sex: How do you know when it feels good?

Lina: It is when it feels good for the girl, I mean, it feels like both want it. You should not force yourself upon the other, then it’s no good. […], but when both want to, so we do it, maybe not every day, but perhaps a few times a week or so, in the daytime.

Lina's self-reflection in the third person provides a perspective on her own experiences and beliefs regarding sexual interaction and highlights the importance of mutual desire and consent in sexual interactions. It became evident that, even before coming close to a sexual situation with someone else, many parameters were involved, which required a great deal of focus from individuals with intellectual disabilities. The participants’ reflections demonstrated a diversity of descriptions of social codes that could lead to being in a sexual situation. For some participants, these interpersonal scripts (Gagnon and Simon, 2017) involved receiving and comprehending verbal and visual cues from their partner. This decision-making process could also be influenced by the element of time since it could take longer to decide about sexual consent. In addition to the participants' emotional state, stress played a central role in shaping their sexual experiences. Some described that stress negatively impacted the desire for sex and that it hindered some respondents from feeling engaged in sexual intercourse as frequently as their partners. Others described how stress influenced the timing of sexual consent. However, stress did not always affect sexual desire negatively. Even when faced with limited opportunities for privacy, which could potentially cause stress, some individuals appeared to be unaffected by this and were able to instantly determine their attraction to someone based on just a few words and to take proactive steps to arrange for privacy. For example, Britt shared how a comment from her partner led her to act.

He said something, and then we did it. I locked both doors, so, ah, I [we] did it. We were at a camp.

Despite Britt being emotionally prepared for a sexual encounter, the physical environment may not always be conducive. For some of the participants, the combination of intellectual disability and daily form played a decisive role in their sexual perceptions, enabling or preventing them from acting on their desires. For example, this could mean that they wanted to have sex, but did not act on it due to fatigue. It could also be linked to a variety of sensitivities, such as being sensitive to tactile stimulation.

Participants also reflected on being young and feeling the urge of sexual desire but lacking sexual experience, which led to uncertainty in the sexual situation. Eric shared the following:

She wanted sex, naked sex. I wanted it too, but I told her that I don’t want to knock you up (…) And I wanted to use a condom, but she said no. And we had [sex], the trunk [penis] didn’t come inside her, luckily enough (Eric)

Even if there was consent, the sexual situation in the above quotation itself seems to have consisted of parallel, unspoken intentions. Eric disclosed in the interview that he felt pressured by his partner's desire to conceive during their sexual encounter, adding complexity to the situation. Despite Eric's intention to use a condom to prevent unwanted pregnancies, his partner's wish to become pregnant created tension and blurred the lines of sexual consent, contributing to Eric's distress.

Having intellectual disabilities may also constitute a hindrance to forming a loving long-term relationship. In the sexual act, their interpersonal scripts (Gagnon and Simon, 2017) differ because her partner wants casual sex and Judith perceives sex as the beginning of a relationship. Judith described the feeling of being single and longing for a relationship, but that her intellectual disabilities were standing in the way:

I think my intellectual disability is affecting me. It is hard because you don’t know how to explain to someone else that you have a disability. But sometimes I can feel like, I am just good enough for sexual contact, but nothing else.

In the consent process, some of the younger adults with intellectual disabilities attempted to modify their sexual codes, norms, and signals based on their cognitive ability and sexual knowledge. This adaptive behavior presented a significant challenge, particularly for individuals who lacked prior sexual experiences, which could potentially influence the development of their future sexual scripts. Some described how they believed this was the appropriate way to engage in sexual activity. Others described lacking the vocabulary to describe their experiences, which resulted in a delay before they discussed them with anyone. Trusting one's partner was a key factor in wanting to indulge in a sexual situation, according to those interviewed. This trust was interpreted as involving consensus in a sexual activity, and it might involve their partner also having intellectual disabilities. Having a partner with intellectual disabilities was described as comforting, as there was then a mutual understanding that enabled them to live out their sexuality.

The sexual situation could be expressed in terms of bodily sensations, having a pleasant time, or being able to relax. Tom was living at home and had previous positive experiences of sexual relationships. He described how he could anticipate when there would be a sexual activity. Tom shared an experience of when he had met up with an old girlfriend from college in a bar, for whom he still felt affection. Even though progressing from meeting up to being in a sexual situation had gone quickly for Tom, he seemed to have interpreted the sexual codes and had achieved a pleasant sexual experience:

By some coincidence, we met up at a bar in town. So, we just went home to her [and had sex]. It happened very fast. She liked me a lot. She was very forward, and I liked that.

Sexual self-awareness varies, but is also something important for the participants, and something that affects their sexual experience. For Tom, previous positive sexual experiences had boosted his sexual self-awareness. This brings us to the way the participants communicate in sexual situations.

### Sexual communication and the necessity of clarification

For individuals with intellectual disabilities, their cognitive capacities influence their abstract thinking, which makes it necessary to contextualize sexual situations (Foley, 2018; Stoffelen et al., 2018). Thus, in the context of sexual communication, the imperative need for clarification plays a pivotal role in consensual sexual interactions for young adults with intellectual disabilities. Sexual communication of this kind encompasses both verbal and nonverbal cues. Depending on their age, their life experience, and their cognitive understanding, the participants used different strategies to comprehend the sexual interaction. Clarification involves both refining the language used and establishing a mutual understanding of the context within a sexual situation. Achieving this may involve open conversations with partner(s) either before or during the sexual encounter, which contributes to a sense of having control over the situation. For many of the participants, it becomes clear that a sexual agency emerges despite the need to adhere to the predetermined script. A few of the participants had experience with BDSM (bondage, dominance or discipline, sadism, and masochism) and used elements as strategies to predict the sexual situation and to feel safe. Consent is a central aspect of BDSM and is often referred to as safe, sane, and consensual (Carlström, 2017). Judith exemplified how she used explicit verbal communication to ensure that the sexual situation was based on sexual consent**, “**We use this as a stop-word [holds up her hand]. If it’s no good, so stop, so to speak.”

The digital arena was another place where strategies were employed to communicate sexual consent. Taking the time to first get to know potential partners digitally could lead to being mentally prepared, comfortable, and secure enough to have sex when meeting up in real life. Daisy described how she had met her partner online and how they spent a long time writing to each other and getting to know each other well, so that “Already on the first night when we met, we slept together and somehow it did not feel strange.”

Even if the cultural script and its norms telling us how long we are supposed to wait before having sex with someone for the first time played a part, the interpersonal script was built up online, so that when meeting up in the way described by Daisy, the intrapsychic script shortened the distance to having sex and this ended up being a positive sexual experience (Gagnon and Simon, 1986, 2017). The understanding of what both want and agree to is central when it comes to mutual sexual consent, but also the ability to anticipate the content of a sexual activity is received as something positive. The result also described the interactions between participants and their partners the way different body signals were linked to arousal and how they were perceived as a “yes” or “no”.

Nellie had been in a relationship with her boyfriend for many years. She expressed her boundaries clearly when she was in sexual situations. In Nelly’s description below, it becomes apparent how the different sexual scripts intersect. The sexual communication that took place at the interpersonal level created a sexual consensus between both Nellie’s and her partner´s intrapsychic scripts (Gagnon and Simon, 2017).

Nellie: He always asks before, do you want sex, and so he asks how I want it. Of course, I asked him too.

Interviewer: Why do you think that is important?

Nellie: Mm, kind of consent.

Interviewer: If you have sex and maybe it doesn’t feel good in the middle of it.

Nellie: But then you must say.

A similar type of sexual communication was described by Bibbi. She spoke of the sexual arousal intensifying mutually, step by step, and then leading into a sexual act. The description below also describes how this was communicated with her partner by reading body signals, or in Bibbi’s own words:

We normally chill a bit at first and then, well, sort of touch each other to arouse the other, until we both feel [whistles]. Something is going on here! Then it becomes rougher, and then it is bang-bang [demonstrates a sexual act with her hands].

It is evident that the individuals themselves played an active part in creating a positive consensual sexual situation with their partner, but also that they used different strategies to control this. Yet, this is a thin line for young adults with intellectual disabilities to balance, which brings us to the final theme.

### Striving for a balance

Striving for a balance was a continuous process that was evident across a spectrum of sexual experiences, spanning from positive to negative encounters. Participants described diverse strategies for maintaining this balance, adapting their approaches based on the specific context and their cognitive abilities.

The participants’ reflections about sexual situations included a diverse range of situations, such as their partner falling asleep whilst having sex, and the uncertain feeling in the middle of a sexual situation when the arousal is gone but one continues to have sex. For some of the participants, negative experiences occurred when chatting online, such as sexual harassment. For others, their sexual consent had been violated to the extreme, with sexual abuse and rape having taken place.

Negative sexual experiences were noted by both male and female participants and might be part of a situation filled with ambivalence and confusion for the individual. For example, when their partner wanted them to enjoy the sexual situation, but they felt unable to sort out whether they wanted or did not want to engage in having sex. Gunnel, who was in a new relationship at the time of the interview, described a sexual situation in which her body signals had acted quicker than her mind and she had found herself in a sexual situation with her partner without realizing it. Gunnel shared the following:

It just happens, so to speak, and I can think, during it, well, this is not what I want right now, but I don’t know how to say it, so I just get through it, because it’s probably over soon.

The combination of cognitive impairment, overprotection, and lack of sexual education and experience may affect the ability to recognize one's own body's signals in time, which creates confusion in the sexual act. Not knowing whether you want to have sex and how to communicate this in the sexual situation may be described as sexual grey areas (Russell-Miller, 2020) or even sexual abuse.

For young adults with intellectual disabilities who have had a conservative upbringing, sexuality becomes even more restricted. Here, the cultural script and the intrapsychic script are closely interlinked in the sense that the individual has been raised with values about what sex with someone else involves. Tove shared that she had been raised with conservative family values, had past negative sexual experiences, and at the same time lacked positive sexual knowledge, which fits with the idea of the restricted script (Löfgren-Mårtenson, 2013), since the subject had never been brought up other than in a cautionary way. She had entered a relationship with a man but rather wanted a friend. This interpretation could be attributed to a combination of her cognitive disability, characterized by challenges in information processing, and her restricted script (Löfgren-Mårtenson, 2013). In this context, she struggled to comprehend her partner's sexual intentions and lacked the appropriate script to guide her preferred actions. Her interpersonal script also collided with the cultural script, since her intentions were based on the classical view of waiting to have sex until married (Gagnon and Simon, 1986, 2017) while her partner wanted sex before marriage. When asked about how she felt and handled the sexual situation. Tove explained:

I was a bit confused too when someone said, yes, but you wanted this. […]. First, one second, no I don’t want to, and then when he says “Yes you want to! Yes, you want to!”. Then I thought, does it show on me that, I mean, is it something that can be seen from looking at me, that maybe I want to, but I don’t want to.

Similar experiences could be noted by those participants who had other experiences of negative sexual situations. These might involve reflections about participating in a sexual situation that might afterward be noted to have been undesirable. A few of the participants shared that they had expressed feeling pain during sex, but this had not always been respected by their partner. This can be exemplified by Lisa, who had previously had positive sexual experiences with another partner. Her intrapsychic script involved an expectation about how the sexual situation should be and feel, but when she was in the sexual situation, the intrapersonal script did not match (Gagnon and Simon, 2017).

Lisa: We slept together, think it was, eh once.

Interviewer: Yes, and then he was rough, you said.

Lisa: Yes! It hurt. I told him, please, please be gentle now.

Interviewer: Yes, and then what happened?

Lisa: I couldn’t sit down for a week.

There were different descriptions of negative sexual experiences. For some, it included experiences of sexual coercion. For others, it was sexual harassment, and a few had experiences of rape. For many of the participants, the abuse had taken place within a relationship when they were young and had no previous sexual experiences. The participants who had experienced sexual violence shared feelings of self-blame, and for some, the violence had resulted in mental health issues. Lina reflected on her experiences:

It was after I came home from school. And then he came in, just in pants and cap. And then you thought, I should have got an idea that something is about to happen. My warning bell rang, but I didn’t take any notice. Instead, I got undressed and only had my vest and knickers on. And then, after a few hours, I began to feel it hurt, that it began to hurt down there. And then he lay on top of me, completely naked when I woke up because I was in pain. And I thought that was such a shitty thing to do.

Among those participants who had experienced sexual violence, many described how they had been active sexual agents in the way they dealt with the situation they had experienced. This could happen in the actual rape situation, as exemplified by Lina:

So I tried to push him away, but I could not because of my muscle disease. That’s why I called help! Help! Then Benny and Johnny [friends] came and pulled him off me.

Lina’s agency was not just present in the actual rape situation but could rather be seen as something ongoing. After the rape, she reported the incident to the police, and the perpetrator was prosecuted and convicted. In addition, she had formed a new relationship, which she described as being built on love and respect.

The process from being sexually abused to moving on could also be seen as a form of sexual agency (Löfgren-Mårtenson, 2020a). This was noted by several of the participants, who described how they, with the support of others, gained strength and got through their experiences. For some, this process also led them to join various organizations. Altogether, this seemed to have had some positive impact on their self-esteem, for example by communicating their reflections on their own negative experiences to the public using social media or by giving seminars as Felicia, described:

I do think, as an influencer, now I´m only talking about myself as an influencer, not as the private Felicia, but more as an influencer and from my influencer role. I do believe it is very important that you talk about this. That you can stand up for your own history.

Sharing experiences with others seemed to be a very important strategy for processing past negative sexual experiences e.g., sexual abuse. Sharing one’s experiences with others may be seen as a form of self-advocacy that leads to empowerment (Löfgren-Mårtenson, 2020b). For those young adults with intellectual disabilities who had been subjected to sexual violence, their sexual scripts (Gagnon and Simon, 2017) had been damaged. However, these scripts are dynamic, and with the aid of various strategies, and time, the victims were able to regain their scripts.

## Discussion

This study aimed to gain an in-depth understanding of young adults with intellectual disabilities' sexual experiences and perceptions of sexual consent. The study's findings indicate that sexual consent and its content are central to how young adults with intellectual disabilities reflect on their sexual experiences with others, and this permeated all four themes. The use of sexual script theory (Gagnon and Simon, 2017) has proven helpful in understanding the complexity of layers involved in a sexual situation and how the sexual acts themselves are different for young adults with intellectual disabilities. In line with Löfgren-Mårtenson´s research (2013), the current study’s results show that participants are influenced by restrictive scripts. These scripts are seen to negatively affect the possibilities for sexual consent, but the results also reveal an agency, a power and a clarity among the participants that promotes their possibility of sexual consent.

The outcomes indicate that the sexuality of young adults with intellectual disabilities becomes public because of the lack of private space and is supervised by parents or caregivers. The sociologist Gisela Helmius has emphasized that society is always a third participant whenever two people have sex (1990). Society is characterized by power structures and norms regarding sexuality, which are dynamic (Foucault, 1998; Weeks, 2010), and as was observed in the theme *Setting the sexual scene*, young adults with intellectual disabilities must overcome many more obstacles than their general population peers to create a private sexual scene. However, adapting in this way can require so much focus that they might forget or not have the time to sexually consent. The results from the study show that there is a fine balance between on the one hand the way professionals and parents can get in the way of young adults’ ability to form sexual relationships and create sexual situations with other people, and on the other hand, the support that young adults with intellectual disabilities need due to their lack of sexual knowledge.

The results illustrate how sexual acts were based on individualistic needs and could differ based on prior sexual experiences. There was thus diversity among the sexual experiences of young adults with intellectual disabilities. As was seen in the theme, *Sexual self-awareness*, there was also a need to keep track of the partner’s sexual intentions. By using specific strategies, the participants facilitate a connection between themselves and their partners. Their positive experiences could be seen as entailing consensual sexual interactions. The experience of sexual situations that one may not understand or like is consistent with McCarthy's (1999) previous research on sexuality among women with mild to moderate intellectual disabilities, but in the current study, the results showed that young male adults with intellectual disabilities can also find themselves in sexual situations of this kind. Even though many of the participants had sexual desires and urges, sexual consent was part of a process that for some could be difficult to comprehend, which may in turn be linked to sexual vulnerability (Hollomotz, 2012). Part of this may be because of sexual inexperience or due to poor sex education (Jahoda and Pownall, 2014; Lukkerz, 2023), but it may also be linked to an inability to absorb this information when it is provided at a special school, due to the professionals not being adequately trained to meet individual needs and the information provided being based on sexual risks (Frawley and Wilson, 2016; Löfgren-Mårtenson, 2012; Schaafsma et al., 2017). Certain research indicates that young adults with intellectual disabilities often acquire knowledge about sex and relationships from 'informal' sources, including porn sites, online platforms, and chat sites (Löfgren-Mårtenson, 2020b). This exposure can lead to distorted perceptions about the nature of sex and relationships (Eastgate et al., 2011) and reinforce stereotypical gender roles (Retznik et al., 2021). This is especially significant during young adulthood, which is a time of sexual identity exploration. Although the results did not reveal any explicit use of pornographic websites, it was observed that many participants utilize the Internet for purposes related to seeking information, dating, and online communication. This underscores the evolving landscape of sexual interactions (Löfgren-Mårtenson, 2020b).

The results also demonstrate that sexual knowledge plays an important part in sexual consent, which has also been noted in previous national research (Löfgren-Mårtenson, 2012; Nelson et al., 2020). As shown by the theme *Sexual communication and the necessity of clarification*, sexual knowledge was intricately woven into participants' sexual interactions. Many participants exhibited an awareness about sexual consent and how to communicate it. At the same time, the presence of sexual agency in the findings is a demonstration of individual power. Nevertheless, as with findings from previous international and national research (Löfgren-Mårtenson, 2020a; Nayak, 2016; Schaafsma et al., 2017), several participants lacked a positive understanding of sexual knowledge. Löfgren-Mårtenson's Swedish study (2012) has revealed that young individuals with intellectual disabilities perceive the topic of sexuality as overly abstract and sometimes irrelevant, while it does not relate to their current life phases or situations. These results highlight the need for contextualized sexual education to meet the needs of young adults with intellectual disabilities, for example, the incorporation of teaching subtler sexual codes in the curriculum. This would promote the sexual responsibility of young adults with intellectual disabilities (Nelson et al., 2020; McCarthy et al., 2022). The results from the current study also indicated that lived experience has a significant influence on the communication of sexual consent.

In addition, the results show that young adults with intellectual disabilities use different strategies and forms of sexual agency depending on the sexual situation. This was noted with both positive and negative sexual experiences, as illustrated by the theme *Striving for a balance*. On the one hand, the agency was initially present in a sexual situation, on the other hand, it could also be noted as part of the participants' recovery from experiences of sexual violence when forming new sexual relationships. It became evident that sexual consent and trust were important in these new relationships. Similar findings can be found in previous research (e.g., Fish, 2016), whose study participants still wanted a sexual relationship even after experiencing sexual violence. Moreover, the study revealed that following sexual assault, some participants actively sought and obtained support from relatives and professionals. The study's findings highlight the essential role of tailored support for individuals within this target group who have experienced sexual offenses, as emphasized in recent research by Hollomotz et al., (2023). Gill (2015) argues that individuals with intellectual disabilities are active participants in their sexuality, thus challenging the societal perception of them as sexually vulnerable (Hollomotz, 2012). This counter-image is crucial in promoting the sexual empowerment of young adults with intellectual disabilities, facilitating informed sexual decision-making (Löfgren-Mårtenson, 2020a). The current study shows similar results for the present sample of young adults with intellectual disabilities, e.g., in the way some of the participants took an active part in processing negative experiences and cultivating new ones with a clear understanding of what matters to them.

The findings revealed intricate layers of cultural norms intertwined with sexuality and intellectual disabilities. Situations that may not align with societal norms, including LGBTQ- identities, often result in a need for secrecy, which increases sexual vulnerability. These findings are in line with previous research highlighting various forms of discrimination among lesbians and bisexual women with intellectual disabilities (Stoffelen et al., 2018). Nevertheless, in this study, the existence of sexual agency is revealed using different strategies.

## Implications for practice

This study offers valuable insights from the voices of young adults with intellectual disabilities for the professionals and families who support them. To provide appropriate support for their sexuality, education and training on sexual consent are essential. This includes understanding social skills, sexual boundaries, rights, and effective communication. Promoting autonomy in decision-making is crucial to helping individuals express preferences and set boundaries. Facilitating access to support services such as counseling and advocacy groups would also be beneficial for professionals and families.

## Limitations and strengths

Several limitations associated with the study should be noted. Firstly, there were gatekeeper challenges as it was difficult to gain access to participants. This also affected the recruitment process, as attempts to meet with potential participants before the interview to inform them of the interview did not work as planned. Future studies focusing on the same target group will need to consider alternative methods to overcome the barriers associated with gatekeepers.

Despite the recruitment settings hosting discussions on sexuality and consent, variations were observed in participants' comfort levels in engaging with these topics, underscoring the importance of understanding individual experiences. Furthermore, the study's inclusion criteria, which required participants to demonstrate linguistic and communicative skills, offered valuable insights during the interviews and reflected a strategic selection process. However, the exclusion of individuals with profound communication challenges constitutes a limitation, highlighting a gap in reaching this subgroup. In Sweden, mandatory sexual education for all students ensures a standardized baseline of information, enhancing the significance of the study's focus on individual variations. However, this may limit generalizability to contexts with different educational frameworks. Future research might benefit from comparative analyses across diverse educational backgrounds to assess the impact of varying sexual education levels on the same demographic. Another limitation is the gender imbalance among the participants (17 women, 5 men), which raises questions regarding potential influences such as the researcher's gender, the interview format, or information dissemination methods. While this disparity does not imply a neglect of men's experiences, future research should strive for a more balanced gender representation to enrich the insights obtained from research. It is worth noting that there was no evidence of gender differences in the willingness to discuss sensitive topics during the interviews. Moreover, the involvement of some participants in disability organizations may have had an impact on the results, potentially impacting the generalizability of findings. Nevertheless, 22 interviews provided rich empirical material and demonstrated the participants' interest in sharing their experiences, emphasizing the depth of insight obtained despite the identified limitations.

## Conclusions and future research

As has been mentioned, the process of communicating sexual consent is a complex path, not just for individuals with intellectual disabilities, but also for young adults more generally. But in the case of people with intellectual disabilities, additional factors come into play. Firstly, cognitive impairment makes it even more challenging to interpret the implicit sexual codes and norms embedded in sexual encounters. Secondly, the sexuality of people with intellectual disabilities is inherently linked to societal power structures that are constantly judging their normalcy, thus producing an inferior negotiating position in comparison with the broader population. The results have shown that prior sexual experiences impact the dynamics of sexual consent. For instance, being younger and having limited sexual experiences may involve a risk of negative experiences and abuse. This might manifest as a lack of knowledge or the vocabulary to articulate consent in sexual contexts. Nevertheless, many participants demonstrated mature reflections about this issue and described their agency in both consensual and non-consensual situations and in the context of surviving sexual abuse. Their degree of sexual agency was influenced by the formal and informal support that participants received from others. This underscores the significance of providing appropriate sexual education and support for young adults with intellectual disabilities which can help promote their safety and well-being.

The current study shows that young adults with intellectual disabilities face several challenges, resulting in a range of sexual experiences from positive encounters to abuse. Sexual consent is a complex issue for most young adults. While many young adults with intellectual disabilities adopt strategies for safe sexual activities, our study underscores that securing sexual consent involves additional complexities. Society is responsible for providing young adults with intellectual disabilities with the essential Sexual and Reproductive Health and Rights based on respect for their sexual privacy. In addition, the study provides insights into how societal norms and expectations can impact the sexual experiences of individuals with intellectual disabilities, which can inform broader discussions around disability rights and social justice.

Overall, the findings of this study demonstrate the important implications for improving the quality of life and social inclusion of individuals with intellectual disabilities, particularly in terms of promoting their sexual health and autonomy. Further research is needed to strengthen the fine line between consent and abuse and to better understand the complex dynamics and challenges associated with sexual consent among young adults with intellectual disabilities.
